# Multiphase photochemical reactions as sinks of nanoplastic photodissolution products in aqueous environments: a model study for benzene

**DOI:** 10.1007/s11356-025-36042-x

**Published:** 2025-02-12

**Authors:** Davide Vione, Monica Passananti, Marco Minella, Luca Carena

**Affiliations:** 1 Dipartimento di Chimica, Università di Torino, Via Pietro Giuria 5, 10125 Turin, Italy; 2 Institute for Atmospheric and Earth System Research/Physics, Faculty of Science, University of Helsinki, 00014 Helsinki, Finland

**Keywords:** Two-compartment models, Environmental fate of pollutants, Water photochemistry, Atmospheric photochemistry, Plastic weathering, Toxic contaminants and hazardous compounds

## Abstract

Carcinogenic benzene is the most concerning product of the irradiation of polystyrene nanoplastics in aqueous suspension. Interestingly, benzene formed in water from polystyrene can volatilise to the gas phase or react with aqueous-phase hydroxyl radicals (^•^OH_(w)_) to produce toxic phenol. The persistence of benzene in water would range from some weeks to some months, and the branching ratio between the ^•^OH_(w)_ reaction and volatilisation mainly depends on water depth and the DOC (dissolved organic carbon) concentration. Actually, benzene volatilisation is particularly important in shallow waters (1–2 m depth), or even in relatively deep waters (> 5 m) if the DOC value is high enough (> 5 mg_C_ L^−1^). Aqueous phenol formed from benzene + ^•^OH_(w)_ reacts in turn with ^•^OH_(w)_, the carbonate radical (CO_3_^•–^_(w)_), and the triplet states of chromophoric dissolved organic matter (^3^CDOM*_(w)_) in different proportions, depending on water chemistry. In the gas phase, benzene reacts with ^•^OH_(g)_ to produce phenol, which in turn reacts with ^•^OH_(g)_ and especially with the nitrate radical (^•^NO_3 (g)_). The overall degradation is fast enough for phenol to reach an extremely low steady-state concentration in the atmosphere. However, up to 50% of the initial water-dissolved benzene would produce gas-phase phenol as intermediate compound and, eventually, yield phytotoxic nitrophenols. Among the latter, 4-nitrophenol has strong potential to partition into atmospheric waters and reach back aqueous environments (or soil) via wet depositions. To a lesser extent, similar phenomena would involve the highly phytotoxic 2,4-dinitrophenol.

## Introduction

Micro- and nanoplastics are important emerging contaminants that are increasingly found in practically all environmental compartments as well as biota (microplastics), or suspected to occur at substantial and concerning levels despite analytical difficulties that currently limit a wide assessment of their environmental occurrence (nanoplastics) (Andrady et al. 2022). In particular, micro- and nanoplastics can either be formed by environmental weathering and fragmentation of plastic particles of larger size fractions, or be emitted from objects, consumer products (e.g., cosmetics), and processes (e.g., machine washing of clothes) (Kumar et al. 2020; Salthammer 2022). Interaction with biota is potentially important, in particular for nanoplastics due to their ability to cross cell membranes (Liu et al 2021).

Another feature of micro- and especially nanoplastics is their high surface-to-volume ratio, which enhances reactivity so that the smallest plastic particles are significantly transformed by absorption of sunlight and/or reaction with photochemically produced reactive intermediates (PPRIs) (Bianco et al. 2020). The main PPRIs are the hydroxyl (^•^OH) and carbonate (CO_3_^•−^) radicals, singlet oxygen (^1^O_2_), and the triplet states of chromophoric dissolved organic matter (^3^CDOM*) (Vione and Scozzaro 2019). Interestingly, in the case of nanosized polystyrene, it has been possible to measure a surface area-normalised, second-order reaction rate constant with ^•^OH (Bianco et al. 2020).

Another interesting issue is that sunlight absorption by nanoplastics in aqueous suspension and the reactions with ^•^OH induce the release of dissolved molecules (Zhu et al. 2020). Photodissolution is potentially important because the mere fragmentation of a particle into smaller pieces is not a real removal process of the particle itself (in the case of nanoplastics formation from microplastics, it might even increase the impact on biota; Liu et al 2021). In contrast, an at least partial dissolution of the particle might be the initial step of its eventual disappearance from the environment. Clearly, the attention in this case has to be shifted to the dissolved transformation products, because irradiated plastics might release compounds that have toxic, endocrine-disrupting, or even mutagenic/carcinogenic properties (Burgos-Aceves et al. 2021). Such dissolved compounds could derive from the degradation of the polymer skeleton, or occur as additives/plasticisers as well as transformation products of the latter (Gewert et al. 2015).

To make an instance, some of us have shown that nanosized polystyrene under long-term irradiation with natural sunlight (14 months) can produce a range of dissolved species (Bianco et al. 2020). Among the main ones, formate would account for ~ 9% of the initial polymer mass, acetate for ~ 12%, and benzoate for ~ 1%. Therefore, > 20% of the initial polymer would undergo photodissolution upon exposure to sunlight. Among the dissolved species that have been detected, the most concerning one was carcinogenic benzene (Bianco et al. 2020). For this reason, it is very important to assess the environmental behaviour of benzene released in aqueous solution by the irradiation of polystyrene.

Poorly biodegradable benzene can undergo photodegradation in aqueous solution, but processes such as the direct photolysis or reactions with photogenerated CO_3_^•−^, ^1^O_2_, or ^3^CDOM* can be excluded on the basis of experimental findings (Vione et al. 2006). Indeed, benzene would almost exclusively react with ^•^OH in sunlit waters (it has been used as ^•^OH probe for decades; Vermilyea and Voelker 2009) and its environmental fate is apparently simple, with phenol as the main transformation product (Vione et al. 2006). However, such an apparently simple scenario is complicated by the fact that benzene is also volatile. We have recently shown that water–air partitioning of volatile molecules cannot be ignored when assessing their environmental fate, because volatilisation kinetics may be significant and gas-phase transformation is often very effective (Fabbri et al. 2023).

For this reason, this work has the goal of assessing the fate of benzene in a two-compartment model of the environment (water + atmosphere) and also of understanding the potential for polar gas-phase transformation products to reach back the water environment (or soil) due to partitioning into atmospheric waters, followed by wet deposition. The overall purpose is to assess the environmental behaviour of a concerning phototransformation product of nanoplastics and to provide insight into an approach that might be applied to similar scenarios involving other pollutants.

## Methods

### Photodegradation in water

Photodegradation kinetics of benzene and phenol in water, as well as phenol formation kinetics from benzene, were modelled with the APEX software (Aquatic Photochemistry of Environmental Xenobiotics) (Vione 2020). This software predicts pseudo-first-order photodegradation rate constants upon direct photolysis and indirect photochemistry of contaminants. Direct photolysis occurs when a compound absorbs sunlight and gets transformed as a consequence, while indirect photochemistry entails degradation upon reaction with PPRIs (including most notably ^•^OH, CO_3_^•−^, ^1^O_2_, and ^3^CDOM*). PPRIs are generated upon absorption of sunlight by photosensitisers, which include nitrate (^•^OH source), nitrite (^•^OH source), and CDOM (chromophoric dissolved organic matter, photochemical source of ^3^CDOM*, ^1^O_2_, and ^•^OH) (Remucal 2014; Yan and Song 2014; McNeill and Canonica 2016). Furthermore, CO_3_^•−^ is produced upon oxidation of HCO_3_^−^ by ^•^OH, and upon CO_3_^2−^ oxidation by both ^•^OH and ^3^CDOM* (Canonica et al. 2005). In addition to being photochemically generated, PPRIs are also quickly scavenged by natural water components. In particular, ^•^OH is scavenged by DOM (dissolved organic matter, not necessarily chromophoric), by HCO_3_^−^ and CO_3_^2−^, as well as by Br^−^ in saltwater and seawater. Moreover, CO_3_^•−^ is scavenged by DOM, ^3^CDOM* is mostly quenched by dissolved O_2_ to produce ^1^O_2_ with ~ 50% yield, and ^1^O_2_ is quenched by collision with water. The PPRIs that survive the main scavenging/quenching processes are involved in the degradation of dissolved pollutants and of naturally occurring compounds (Vione and Scozzaro 2019).

APEX takes into account PPRI photogeneration and scavenging/quenching as mentioned above, computing the absorption of polychromatic sunlight by CDOM, nitrate, nitrite, and, where relevant, the pollutant(s). As a consequence, photodegradation kinetics are calculated as a function of sunlight irradiance, water depth, and water chemistry (concentration values of nitrate, nitrite, bicarbonate, carbonate, and dissolved organic carbon, DOC) (Silva et al. 2015, 2019; Vione 2020). APEX assumes well-mixed water bodies under clear-sky conditions, and in these circumstances, it has been shown to predict environmental photodegradation kinetics with good accuracy. APEX predictions include the phototransformation of the phenolic derivatives benzophenone-3 and benzophenone-4 and the aqueous-phase reaction kinetics between toluene and ^•^OH (Vione 2020). Benzene has similar structure as toluene, and, like toluene, it only reacts with ^•^OH (Buxton et al. 1988), while benzophenone-3, benzophenone-4, and phenol all share the phenolic function. APEX is thus expected to suitably predict photodegradation kinetics of both benzene and phenol.

### Benzene volatilisation and gas-phase transformation processes

The volatilisation rate constant of benzene from aqueous environments was predicted with the EPISUITE™ package by US-EPA (US-EPA 2021) on the basis of molecular structure (quantitative structure–activity relationship), as a function of water depth and assuming 0.5 m s^−1^ wind speed as well as 0.05 m s^−1^ current speed (typical lake-water conditions). The gas-phase reaction rate constants of both benzene and phenol with ^•^OH and of phenol with ^•^NO_3_ were taken from the literature. Furthermore, it was assumed a 24-h [^•^OH _(g)_] = 1.5 × 10^6^ cm^−3^ (average of typical daytime values, together with ^•^OH absence during the night) and [^•^NO_3 (g)_] = 5 × 10^7^ cm^−3^ (~1 pptv as 24-h average, considering night-time occurrence and absence in typical daytime) (Finlayson-Pitts and Pitts 2000).

Some of phenol transformation products in the gas phase are polar enough to partition into atmospheric waters (e.g., a cloud), and the partitioning equilibrium of all relevant compounds (including phenol) was considered by using an approach based on the Henry’s law. In particular, the Henry’s law constant can be defined as the ratio between the molar concentration values of a given compound P in atmospheric waters and in the gas phase (Sander 2000): *K*_H_ = *c*_P,w_ *c*_P,g_^−1^. Therefore, at equilibrium, the water concentration resulting from phase partitioning is *c*_P,w_ = *K*_H_ *c*_P,g_. Phase transfer to reach equilibrium occurs when some moles of P (*n*_P_) pass from the gas phase to water and vice versa, which can be related to molar concentrations by knowing the volumes of both the gas phase and atmospheric waters (or their volume ratios, vide infra): *n*_P,w_ = *c*_P,w_ *V*_w_; *n*_P,g_ = *c*_P,g_ *V*_g_.

## Results and discussion

Benzene has been frequently used as ^•^OH probe in irradiated surface-water samples, and the recent attempts to replace it with alternative compounds (e.g., terephthalate) are due to safety concerns rather than to lack of reaction selectivity with ^•^OH (Page et al. 2010). The reaction between benzene and ^•^OH in water has a second-order rate constant *k*_B,•OH(w)_ = 7.8 × 10^9^ M^−1^ s^−1^ (Buxton et al. 1988), while benzene does not undergo direct photolysis or reaction with other PPRIs (CO_3_^•−^, ^1^O_2_, ^3^CDOM*) to a significant extent (Vione et al. 2006). Biodegradability is also quite poor (US-EPA 2021). It is thus safe to assume that benzene would mainly react with ^•^OH in aqueous environments. However, volatilisation from the aqueous phase could also be important and should be taken into account, when considering the environmental fate of benzene (US-EPA 2021). The aqueous-phase reaction between benzene and ^•^OH_(w)_ gives phenol (Ph) with a reported yield *η*_Ph(w)_ = 0.85 (Vermilyea and Voelker 2009). Phenol is poorly volatile (its predicted volatilisation lifetimes are in the order of a couple of years for lake waters with depth *d* = 1 m; US-EPA 2021), but it reacts in water with ^•^OH (*k*_Ph,•OH(w)_ = 1.4 × 10^10^ M^−1^ s^−1^), CO_3_^•−^ (*k*_Ph,CO₃•_-_(w)_ = 1.6 × 10^7^ M^−1^ s^−1^), and ^1^O_2_ (*k*_Ph,__¹__O₂(w)_ = 10^6^ M^−1^ s^−1^) (Buxton et al. 1988; Wilkinson et al. 1995; Wojnárovits et al. 2020). Moreover, a reasonable estimate for the reaction rate constant between phenol and ^3^CDOM* is *k*_Ph,__³__CDOM*(w)_ = 6.4 × 10^8^ M^−1^ s^−1^ (McNeill and Canonica 2016). Note that neither benzene nor phenol absorb sunlight significantly, which rules out their direct photolysis.

Evidence is reported that the photodegradation of phenol could be considerably accelerated at the air–water interface (Kusaka et al. 2021), a phenomenon that affects a very thin layer at the water surface. Actually, enhanced interface reactivity would be more important for atmospheric aerosols than for surface water bodies that have depths in the metre range. Furthermore, the cited findings consider the direct photolysis of phenol under UV-C radiation (Kusaka et al. 2021), which is not environmentally significant. While indirect photochemical reactions could also be accelerated at the interface, the expected degree of enhancement (Anglada et al. 2020) is much lower compared to the direct photolysis of phenol. For these reasons, reactivity at the air–water interface was not considered here.

When reaching the atmospheric gas phase by volatilisation, benzene would mainly react with ^•^OH_(g)_ (*k*_B,•OH(g)_ = 1.9 × 10^−12^ cm^3^ molecule^−1^ s^−1^) and the main reaction product is again phenol (yield *η*_Ph(g)_ ~ 0.5; Volkamer et al. 2002). Gas-phase phenol would mainly react with ^•^OH during the day (*k*_Ph,•OH(g)_ = 3.3 × 10^−11^ cm^3^ molecule^−1^ s^−1^) and with ^•^NO_3_ during the night (*k*_Ph,•NO₃(g)_ = 5.8 × 10^−12^ cm^3^ molecule^−1^ s^−1^) (Atkinson 1986).

**Scheme 1:**
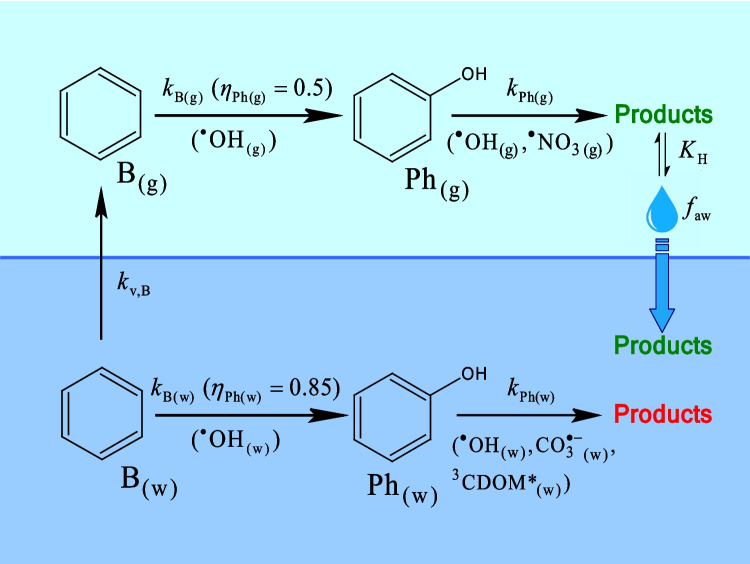
Outline of possible benzene transformation processes in water and in the gas phase. Phenol is highlighted as major transformation product. Note that *k*_B(w)_, *k*_v,B_, *k*_B(g)_, *k*_Ph(w)_, and *k*_Ph(g)_ are all first-order rate constants. *K*_H_ is the Henry’s law constant, while *f*_w_ is the fraction of liquid water in the atmosphere

Except for volatilisation, the mentioned processes are second-order reactions involving transient species in either water or air. These transients are quickly scavenged and reach very low steady-state concentrations; thus, the relevant reactions actually follow pseudo-first-order kinetics. Indeed, each process (benzene photodegradation in, and volatilisation from, water; phenol degradation in water; degradation of benzene and phenol in air) can be described by a first-order rate constant (see Scheme 1).

On the basis of the processes and the rate constants depicted in the scheme, the time trends of the molar concentrations of aqueous-phase benzene (*B*_(w)_), aqueous-phase phenol (Ph_(w)_), gas-phase benzene (B_(g)_), and gas-phase phenol (Ph_(g)_) can be described by the following equations (Fabbri et al 2023):

1 $$\left[{\mathrm{B}}_{(\mathrm{W})}\right]/{\left[{B}_{(\mathrm{W})}\right]}_{\mathrm{o}}={e}^{-({k}_{\mathrm{B}\left(\mathrm{w}\right)}+{k}_\mathrm{V,B})t}$$

2 $$\left[{\mathrm{Ph}}_{\left(\mathrm{w}\right)}\right]/{\left[{\mathrm{B}}_{\left(\mathrm{w}\right)}\right]}_{\mathrm{o}}=\frac{{\eta }_{\mathrm{Ph}(\mathrm{w})}{k}_{\mathrm{B}(\mathrm{w})}}{{k}_{\mathrm{Ph}(\mathrm{w})}-{k}_{\mathrm{B}(\mathrm{w})}-{k}_{\mathrm{v},\mathrm{B}}}[{e}^{-({k}_{\mathrm{B}\left(\mathrm{w}\right)}+{k}_{\mathrm{v},\mathrm{B}})t}-{e}^{-{k}_{\mathrm{Ph}(\mathrm{w})}t}]$$

3 $$\left[{\mathrm{B}}_{(\mathrm{g})}\right]/{\left[{\mathrm{B}}_{(\mathrm{w})}\right]}_{\mathrm{O}}=\frac{{k}_{\mathrm{v},\mathrm{B}}}{\mathfrak{R}\left({k}_{\mathrm{B}(\mathrm{g})}-{k}_{\mathrm{B}(\mathrm{w})}-{k}_{\mathrm{v},\mathrm{B}}\right)}[{e}^{-({k}_{\mathrm{B}\left(\mathrm{w}\right)}+{k}_{\mathrm{v},\mathrm{B}})t}-{e}^{-{k}_{\mathrm{B}(\mathrm{g})}t}]$$

4 $$\left[{\mathrm{Ph}}_{\left(\mathrm{g}\right)}\right]/{[{B}_{(\mathrm{w})}]}_{\mathrm{o}}=\frac{{\eta }_{\mathrm{Ph}(\mathrm{g})}{k}_{\mathrm{v},\mathrm{B}}}{\mathfrak{R}({k}_{\mathrm{B}\left(\mathrm{w}\right)}+{k}_{\mathrm{v},\mathrm{B}})}\left(1+\frac{{k}_{\mathrm{v},\mathrm{B}} {e}^{-{k}_{\mathrm{B}(\mathrm{g})}t}-{\eta }_{\mathrm{g}}{k}_{\mathrm{B}(\mathrm{g})}{e}^{-{k}_{\mathrm{v},\mathrm{B}}t}}{{\eta }_{\mathrm{g}}{k}_{\mathrm{B}(\mathrm{g})}-{k}_{\mathrm{v},\mathrm{B}}}\right){e}^{-{k}_{\mathrm{Ph}(\mathrm{g})}t}$$

where [*B*_(w)_]_o_ is the initial concentration of benzene in water, $\mathfrak{R}$ = *V*_g_/*V*_w_ is the volume ratio between the gas phase and the aqueous phase, and Scheme 1 can be used as reference for each first-order rate constant *k*.

**Fig. 1:**
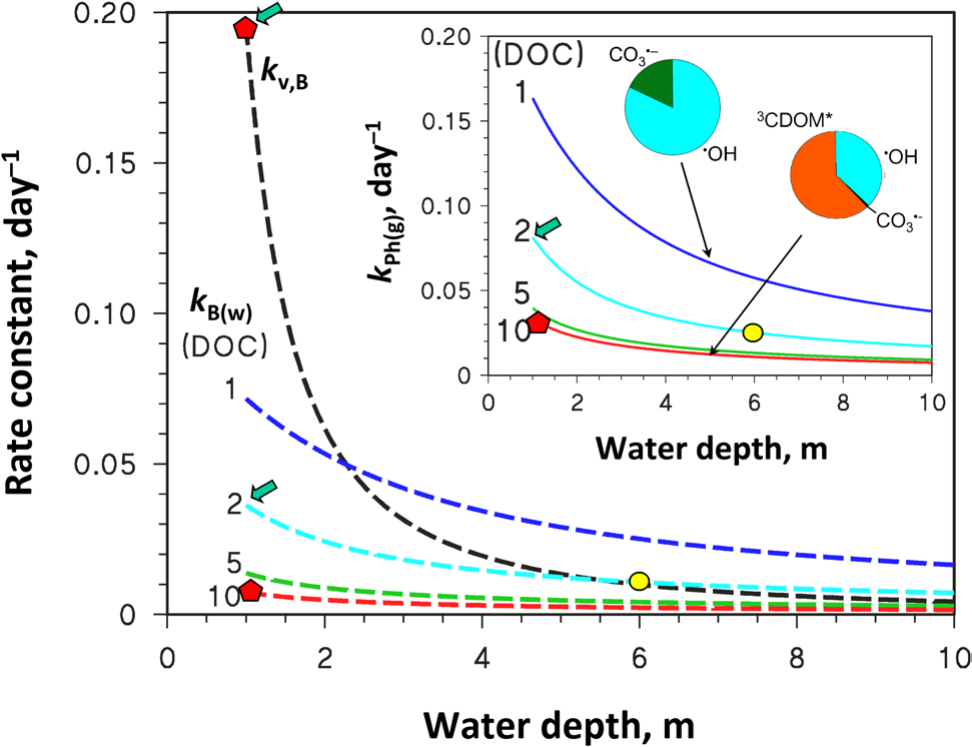
Modelled pseudo-first-order rate constants of benzene removal from water, as a function of water depth in the case of *k*_v,B_, and of both water depth and the DOC in the case of *k*_B(w)_. Note that the numbers near the dashed curves (1, 2, 5, and 10) are DOC values expressed in mg_C_ L^−1^. The insert shows the values of *k*_Ph(w)_ as a function of both depth and DOC (DOC values in mg_C_ L^−1^ are shown near the solid curves). The pie charts show the fractions of phenol undergoing degradation via different photoreaction pathways (^•^OH, CO_3_^•−^, ^3^CDOM*). Other water conditions: 10^−4^ M nitrate, 10^−6^ M nitrite, 10^−3^ M bicarbonate, and 10^−5^ M carbonate. For the calculation of *k*_v,B_, it was assumed a lake with the given depth [m], as well as 0.5 m s^−1^ wind speed and 0.05 m s^−1^ current speed. The arrow, pentagon, and circle highlight different scenarios that will be considered later on

We used APEX to derive the values of the rate constants *k*_B(w)_ and *k*_Ph(w)_ and EPISuite™ to obtain *k*_v,B_ for benzene volatilisation. Note that photoreaction kinetics in water (*k*_B(w)_, *k*_Ph(w)_) are affected by many parameters, among which water depth and the dissolved organic carbon (DOC) play major role (Vione and Scozzaro 2019). Water depth *d* also affects *k*_v,B_ considerably. The calculated values of the mentioned rate constants are shown in Fig. 1, as a function of *d* and DOC (*k*_B(w)_, *k*_Ph(w)_) and of *d* alone (*k*_v,B_). From Fig. 1, it is apparent that all the rate constants decrease with *d*, but the decrease is more important in the case of *k*_v,B_. The rate constant decrease with depth is reasonable because a shallower water body has better contact with the atmosphere, which enhances volatilisation, and is more thoroughly illuminated by sunlight, which enhances photodegradation (Vione and Scozzaro 2019). The reported trends suggest that *k*_v,B_ > *k*_B(w)_ in shallow waters only, unless the water DOC is very high. In fact, *k*_B(w)_ decreases with increasing DOC that measures the dissolved organic matter (DOM), which is a major ^•^OH_(w)_ scavenger. Furthermore, the reaction with ^•^OH_(w)_ is the only benzene photodegradation pathway (Buxton et al. 1988). In the case of phenol, the rate constant *k*_Ph(w)_ decreases with increasing DOC, too, but with an interesting difference: *k*_B(w)_ is about twice higher when DOC = 5 mg_C_ L^−1^ compared to DOC = 10 mg_C_ L^−1^, while the values of *k*_Ph(w)_ are similar in the two cases (see insert in Fig. 1).

Actually, unlike benzene that reacts with ^•^OH_(w)_ only, phenol is degraded by ^•^OH_(w)_, CO_3_^•−^_(w)_, and ^3^CDOM*_(w)_ (and by ^1^O_2__(w)_ to a very minor extent; Wilkinson et al. 1995). Increasing organic matter (DOC) inhibits the ^•^OH_(w)_ and CO_3_^•−^_(w)_ processes through scavenging, but it enhances the ^3^CDOM*_(w)_ reaction that partially offsets the inhibition of the other pathways (Vione and Scozzaro 2019). As shown in the insert to Fig. 1 (pie charts), in the presence of DOC = 1 mg_C_ L^−1^ and with *d* = 5 m, the reactions with ^•^OH_(w)_ (especially) and CO_3_^•−^_(w)_ would be the main phenol degradation pathways. At the same depth, but with DOC = 10 mg_C_ L^−1^, ^3^CDOM*_(w)_ would prevail as the reactive species for phenol degradation. Similar considerations hold for the same DOC values at other depths, because water depth strongly affects kinetics but has lesser influence over the degradation pathways.

As mentioned before, benzene would partly undergo volatilisation to the gas phase, especially from shallow waters. Once in the atmosphere, benzene would react with ^•^OH_(g)_ with pseudo-first-order rate constant *k*_B(g)_ = *k*_B,•OH(g)_ [^•^OH_(g)_]. When considering an average [^•^OH_(g)_] = 1.5 × 10^6^ molecules cm^−3^ during a 24-h day (Lelieveld et al. 2016) and the value of *k*_B,•OH(g)_ reported before, one gets *k*_B(g)_ = 0.13 day^−1^. In turn, phenol formed from benzene would react with both ^•^OH_(g)_ (day) and ^•^NO_3 (g)_ (night), with *k*_Ph(g)_ = *k*_Ph,•OH(g)_ [^•^OH_(g)_] + *k*_Ph,•NO₃(g)_ [^•^NO_3 (g)_] (Atkinson 1986; Bolzacchini et al. 2001). With the same [^•^OH_(g)_] as above, and even with a relatively low [^•^NO_3 (g)_] = 5 × 10^7^ molecules cm^−3^ (~1 pptv as 24-h average; Khan et al. 2015), one gets *k*_Ph(g)_ = 15 day^−1^ that is largely due to the reaction with ^•^NO_3 (g)_.

**Fig. 2:**
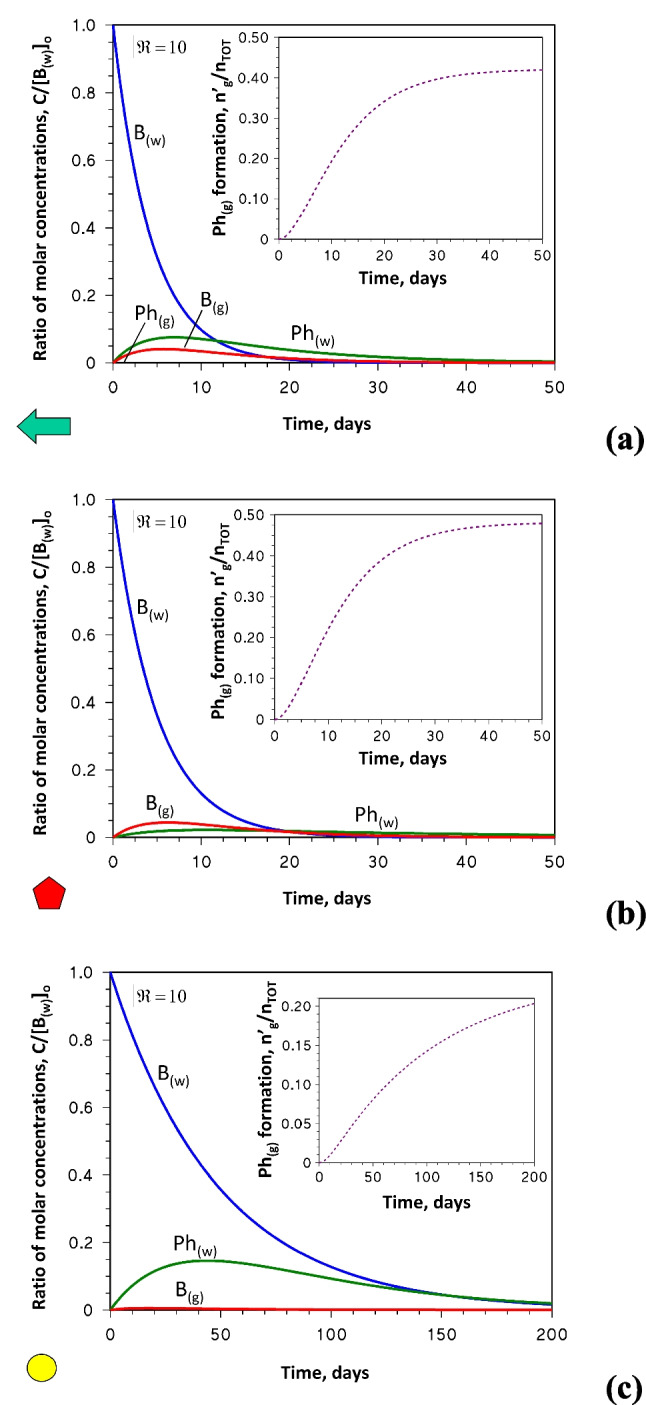
Time trends of benzene (B) and phenol (Ph) in both water (w) and gas (g) phases. Note the negligible concentration of Ph_(g)_ and the week-to-month lifetime of *B*_(w)_. The figure insert shows the ratio between the moles of formed Ph_(g)_ and the initial moles of benzene in water (after formation, Ph_(g)_ would be transformed very quickly). The arrow **a**, pentagon **b**, and circle **c** in the bottom left corner of each plot are a reminder that the water conditions investigated here are those highlighted by the same symbols in Fig. 1. The quantity on the Y-axis is the ratio between the molar concentration C of each compound, in either water or the gas phase, and the initial concentration of benzene in water, [B_(w)_]_o_. Therefore, C/[B_(w)_]_o_ stands for [B_(w)_]/[B_(w)_]_o_, [Ph_(w)_]/[B_(w)_]_o_, [B_(g)_]/[B_(w)_]_o_, or [Ph_(g)_]/[B_(w)_]_o_

When starting from an aqueous phase with *d* = 1 m and DOC = 2 mg_C_ L^−1^, plus 10^−4^ M NO_3_^−^, 10^−6^ M NO_2_^−^, 10^−3^ M HCO_3_^−^, and 10^−5^ M CO_3_^2−^, which are reasonable values for surface freshwaters (Vione et al. 2006; these conditions are highlighted with arrows “⬅” in the plots of Fig. 1), one gets *k*_B(w)_ = 0.036 day^−1^, *k*_Ph(w)_ = 0.081 day^−1^, and *k*_v,B_ = 0.20 day^−1^. By adding the gas-phase reactivity (*k*_B(g)_ = 0.13 day^−1^ and *k*_Ph(g)_ = 15 day^−1^), it is possible to use Eqs. (1–4) to assess the time trends of benzene and phenol in both water and air. The relevant time trends are shown in Fig. 2a, with reference to the initial benzene concentration in the aqueous phase, [B_(w)_]_o_.

Note that Eqs. (3, 4) depend on the ratio *ℜ* = *V*_g_/*V*_w_ between the gas and the water phases. In this case, *V*_g_ represents the volume of the gas phase that benzene could reach by diffusion or convection in a reasonable time period, after leaving the water surface. The choice of the *ℜ* value affects the concentrations [B_(g)_] and [Ph_(g)_]: the larger the *ℜ*, the larger the gas-phase volume and, therefore, the degree of dilution of the relevant compounds. As a consequence, [B_(g)_] and [Ph_(g)_] decrease with increasing *ℜ*. However, the value of *ℜ* does not affect the moles of benzene that leave water by volatilisation or the ratio between [B_(g)_] and [Ph_(g)_]. Our choice of *ℜ* = 10 was mainly motivated by the need to plot [B_(w)_], [Ph_(w)_], [B_(g)_], and [Ph_(g)_] on the same graph, to secure plot readability. The discussion that follows is independent of the actual choice of the *ℜ* value.

Under the water conditions highlighted as “⬅” (Fig. 2a), most benzene would undergo volatilisation rather than reaction with ^•^OH_(w)_ (*k*_v,B_ ~ 5.5 *k*_B(w)_). However, the *V*_g_/*V*_w_ ratio more than compensates for that and leads to [Ph_(w)_] > [B_(g)_]. It is also *k*_B(g)_ > *k*_Ph(w)_, for which reason B_(g)_ disappears faster than Ph_(w)_. A further issue is that [Ph_(g)_] is extremely low. Actually, [Ph_(g)_] arises from relatively slow B_(g)_ degradation (*k*_B(g)_ = 0.13 day^−1^, *η*_Ph(g)_ = 0.5) and quite fast transformation of Ph_(g)_ itself (*k*_Ph(g)_ = 15 day^−1^), thereby satisfying the steady-state condition quite well. However, low to negligible [Ph_(g)_] does not mean that Ph_(g)_ is not formed at all.

Consider the ratio between the moles of generated Ph_(g)_ (*n*’_g_) and the initial moles of *B*_(w)_ (*n*_TOT_):

5 $$\frac{{n}_{\mathrm{g}}^{\prime}}{{n}_{\mathrm{TOT}}}=\frac{{c}_{\mathrm{g}}^{\prime}{V}_{\mathrm{g}}}{{c}_{w}{V}_{w}}=\frac{{c}_{\mathrm{g}}^{\prime}}{{c}_{w}}\mathfrak{R}$$

Note that *c′*_g_ = [Ph_(g)_] is proportional to *ℜ*^−1^ (Eq. 4); thus, the ratio *n′*_g_ / *n*_TOT_ is actually independent of *ℜ*. The cumulative concentration value of the gas-phase phenol that is formed (*c*′_g_) can be obtained from Eq. (4) by neglecting phenol degradation in the gas phase (${e}^{-{k}_{\mathrm{Ph}(\mathrm{g})}t}$ = 1). The resulting values of *n*′_g_ / *n*_TOT_ are plotted in the insert to Fig. 2a, showing that around 40% of the initial *B*_(w)_ would end up as Ph_(g)_ and, most notably, as Ph_(g)_ transformation products.

Compared to the results reported in Fig. 2a (shallow water and low DOC, scenario “⬅”), the conditions characterised by shallow water (*d* = 1 m) and high DOC (10 mg_C_ L^−1^) (scenario “⬟”, Fig. 2b) are even more favourable to benzene volatilisation. First of all, shallow water enhances volatilisation processes (see Fig. 1) and, from this point of view, the “⬅” and “⬟” scenarios are fully equivalent. However, high DOC in “⬟” means low [^•^OH_(w)_], which slows down the reaction of aqueous-phase benzene to produce Ph_(w)_. If the B_(w)_ + ^•^OH_(w)_ reaction is inhibited, the competing volatilisation process (B_(w)_ → B_(g)_) is correspondingly enhanced. For this reason, benzene volatilisation is highly favoured in the “⬟” scenario, which also favours the formation of Ph_(g)_ (Fig. 2b).

In contrast, a relatively low degree of benzene volatilisation is observed in deeper waters (*d* = 6 m, scenario “⬤”, Fig. 2c). In this case, slow benzene volatilisation induced by the water depth favours the B_(w)_ + ^•^OH_(w)_ reaction, with higher production of Ph_(w)_ (which reaches higher values compared to the other scenarios, see Fig. 2c) and lower B_(g)_ formation. In this case, just 20% of the initially occurring B_(w)_ moles would produce Ph_(g)_, as shown in the insert to Fig. 2c.

Based on the previous discussion, benzene volatilisation is inhibited in deep waters and enhanced in shallow ones, especially if the DOC is high. The volatilisation process induces the formation of Ph_(g)_ from B_(g)_ + ^•^OH_(g)_. Interestingly, most Ph_(g)_ reacts with ^•^NO_3 (g)_, and phytotoxic and potentially mutagenic nitrophenols are among the main Ph_(g)_ transformation products in this reaction. The most relevant compounds are 2-nitrophenol (2NP), 4-nitrophenol (4NP), as well as 2,4-dinitrophenol (2,4DNP) (Bolzacchini et al. 2001). Nitrophenols undergo gas-phase photolysis, degradation by reaction with ^•^OH_(g)_, and partitioning into atmospheric waters (Atkinson 1986; Finlayson-Pitts and Pitts 2000; Bejan et al. 2007, 2020). The latter process is described by the Henry’s law constant that is here defined as the ratio of molar concentrations in atmospheric waters (*c*_aw_) *vs*. the gas phase (*c*_g_). It reads as *K*_H_ = *c*_aw_ (*c*_g_)^−1^ (Guo and Brimblecombe 2007). Assume *V*_aw_ as the volume of the atmospheric aqueous phase, and *V*_g_ as the volume of the gas phase. Because *V*_g_» *V*_aw_, the fraction of liquid water in the atmosphere is *f*_aw_ = *V*_aw_ (*V*_g_)^−1^. Starting from the above formulation of *K*_H_ and considering phase transfer, the following ratio is obtained between the moles of a compound in the atmospheric aqueous phase (*n*_aw_ = *c*_aw_ *V*_aw_) and the total number of moles of the same compound in the atmosphere (*n*_T,a_ = *c*_aw_ *V*_aw_ + *c*_g_ *V*_g_):

6 $$\frac{{n}_{\mathrm{aw}}}{{n}_{\mathrm{T},\mathrm{a}}}=\frac{{K}_{\mathrm{H}}{f}_{\mathrm{aw}}}{1+{K}_{\mathrm{H}}{f}_{\mathrm{aw}}}$$

**Fig. 3:**
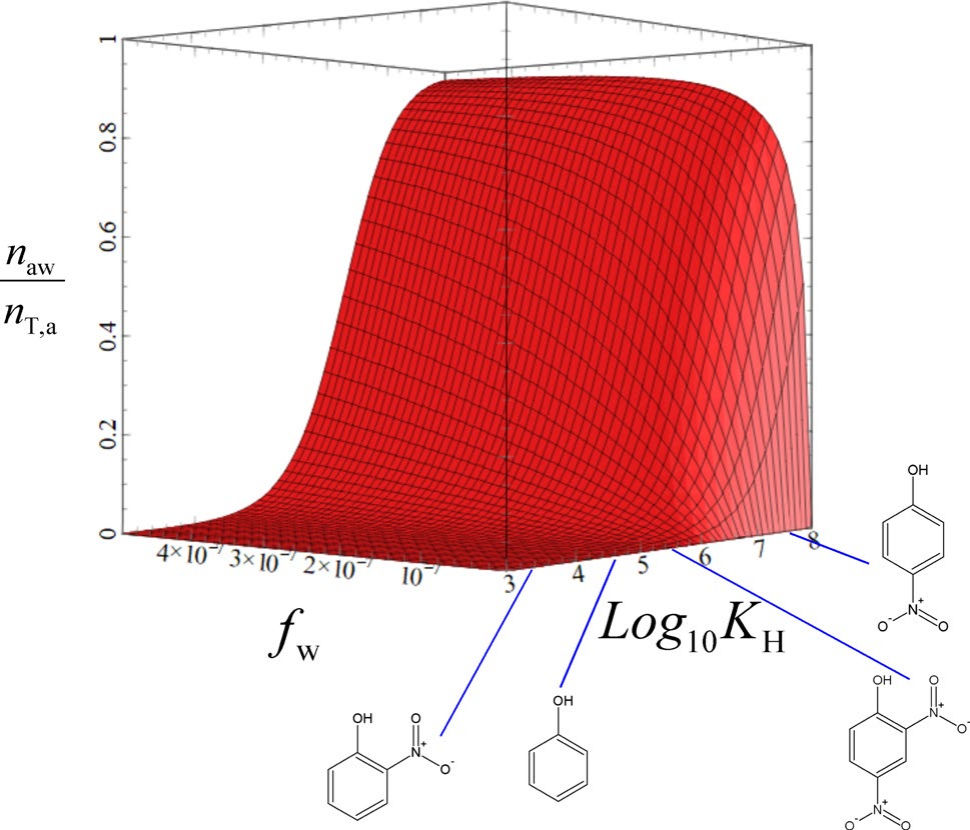
Plot of *n*_aw_/*n*_T,a_ as a function of Log_10_*K*_H_ and of *f*_aw_ varied between 0 (no liquid water in the atmosphere) and 5 × 10^−7^ (cloud). Note that the *f*_aw_ axis goes from right to left. The Log_10_*K*_H_ values of Ph, 2NP, 4NP, and 2,4DNP are highlighted

Figure 3 reports the ratio *n*_aw_/*n*_T,a_ as a function of *K*_H_ (Log_10_*K*_H_ is used here for plot readability issues) and of *f*_aw_. The maximum value of *f*_aw_ (5 × 10^−7^) is referred to the liquid water content in a cloud (Oh et al. 2018). The Log_10_*K*_H_ values of Ph, 2NP, 4NP, and 2,4DNP (Sander 2000) are also highlighted, showing that the tendency to undergo partitioning into the atmospheric aqueous phase follows the order 4NP > 2,4DNP > Ph > 2NP. Therefore, Ph and especially 2NP would mostly remain in the gas phase at any *f*_aw_ value, while 2,4DNP would partition to the water phase if *f*_aw_ is high enough. As far as 4NP is concerned, it would mostly occur in atmospheric waters unless *f*_aw_ → 0; in the latter case, however, 4NP might still be found on airborne particulate matter (Bejan et al. 2020). The low water affinity of 2NP is accounted for by the intra-molecular hydrogen bond that is formed between its OH and NO_2_ groups occurring in *ortho* position, which limits interaction of either group with water molecules. A similar intra-molecular hydrogen bond also explains the lower water affinity of 2,4DNP compared to 4NP, although the NO_2_ group in *para* position increases the water solubility of both compounds.

Therefore, among the transformation products of Ph_(g)_, phytotoxic 2,4DNP and, to a higher extent because it is more soluble in water, 4NP could reach back the ground by liquid deposition. Considering that the eventual occurrence of nitrophenols in the atmospheric aqueous phase would be initially triggered by benzene volatilisation (B_(w)_ → B_(g)_), it is evident that conditions favouring the volatilisation process would be conducive to nitrophenol formation and water partitioning. Most favourable conditions to volatilisation imply shallow water with high DOC, as per the “⬟” scenario previously discussed (Fig. 2b).

## Conclusions

Benzene is a concerning phototransformation product of polystyrene, due to its known carcinogenicity. The persistence of benzene in aquatic environments would range from weeks to months depending on water conditions, and relatively fast elimination would be observed in shallow waters with low DOC. Water-dissolved benzene would either react with ^•^OH_(w)_ or undergo volatilisation to the gas phase. The ^•^OH_(w)_ reaction is inhibited as the water depth and/or the DOC increase, and volatilisation is negatively affected by depth to a higher extent than the ^•^OH_(w)_ process. Therefore, benzene volatilisation would mostly be important in shallow waters, unless the DOC value is very high.

Phenol is the main product of both the ^•^OH_(w)_ (85% yield) and ^•^OH_(g)_ (50% yield) reactions of benzene. In the water phase, phenol would mainly react with ^•^OH_(w)_, CO_3_^•−^_(w)_, and ^3^CDOM_(w)_. The ^•^OH_(w)_ process might lead to detoxification, while CO_3_^•−^_(w)_ and ^3^CDOM_(w)_ oxidise phenol to the phenoxyl radical that eventually yields dimeric species. The latter are more toxic than monomeric phenol towards aquatic organisms (Vione and Scozzaro 2019; Vione 2020).

Gas-phase phenol would react very quickly with both ^•^OH_(g)_ during the day and, especially, ^•^NO_3 (g)_ during the night. The latter is the main transformation process of atmospheric phenol and produces phytotoxic nitrophenols with relatively high yields. Among these nitroderivatives, the *para* isomer (4-nitrophenol) is particularly prone to partitioning to the atmospheric water phase, from which it could reach back soil and surface waters via precipitation and other wet deposition phenomena. The same issue, although to a lesser extent, would apply to phytotoxic 2,4-dinitrophenol. Conversely, 2-nitrophenol would mainly remain in the gas phase, where it could undergo direct photolysis and ^•^OH_(g)_ reaction (Bejan et al. 2007).

Carcinogenic benzene would thus be transformed into mostly toxic species that, due to their usually low concentration levels, would presumably hardly reach toxicity thresholds in the relevant environmental compartments. Overall, environmental attenuation of benzene could take place, or transformation with change in target organisms (e.g., plants in the case of 2,4-dinitrophenol). Furthermore, it is suggested that phase transfers from aquatic environments to the atmosphere (and back to the ground upon precipitation) could be quite common in the environmental fate of several organic pollutants. Therefore, two-compartment models like that used here can be very useful to assess the environmental fate of contaminants.

## Data Availability

Data will be made available on request.
